# Recent Advances in the Diagnosis and Management of High-Risk Cutaneous Squamous Cell Carcinoma

**DOI:** 10.3390/cancers14143556

**Published:** 2022-07-21

**Authors:** Clio Dessinioti, Alexander J. Stratigos

**Affiliations:** 1st Department of Dermatology, Skin Cancer and Melanoma Unit, Andreas Sygros Hospital, University of Athens, 16121 Athens, Greece; alstrat2@gmail.com

**Keywords:** high-risk, cutaneous squamous cell carcinoma, diagnosis, recurrence, metastasis, adjuvant, treatment

## Abstract

**Simple Summary:**

Cutaneous squamous cell carcinoma (cSCC) is the second most common skin cancer. Although most cSCCs are effectively treated with surgery, there are some tumors at higher risk for relapse, progression to nodal metastasis, or, rarely, death. An important issue concerns the early diagnosis and appropriate treatment of these few high-risk cSCCs, as they are associated with poorer prognosis and may more frequently progress to advanced cSCCs. This review discusses the characteristics of high-risk cSCC and how to identify and manage patients with these tumors.

**Abstract:**

High-risk cSCC is defined as invasive cSCC staged as N0 (without detectable regional lymph nodes) and M0 (without distant metastasis), that has features associated with a higher risk of poorer prognosis. The focus of this review is on the recent advances in the diagnosis and management of high-risk cSCC. The interest in high-risk cSCC relies on its higher risk of progression to advanced cSCC, as it represents the main pool of cSCCs that give rise to advanced tumors. Assessment of the risk is thus particularly relevant for common cSCC to identify the few with a high-risk risk of local recurrence, metastasis, or disease-specific death among all other low-risk tumors. The timely diagnosis and effective treatment of high-risk cSCCs may halt their further progression and aim to prevent and lower the incidence of advanced cSCCs. Clearance of the tumor with negative surgical margins is the main goal of surgery, which is the primary treatment of cSCC. It seems that it is difficult to discern the group of high-risk cSCCs that may benefit from adjuvant RT, as a universal beneficial effect for a cSCC with any high-risk factor which was resected with clear surgical margins has not been established. In the case of a high-risk cSCC with positive margins after surgery, and re-excision not feasible, post-operative radiotherapy is performed when possible. Recommendations on further management are discussed. Regarding the follow-up of patients diagnosed with high-risk cSCC, factors to consider regarding the frequency and intensity of the follow-up schedule include the risk and possible time of occurrence of metastasis from cSCC.

## 1. Introduction

**Figure 1:**
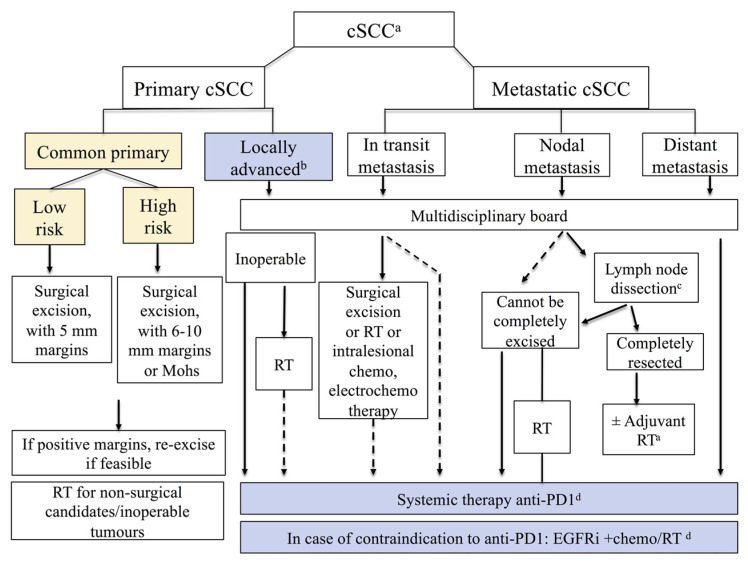
Classification of invasive cSCC in common primary cSCC (high-risk or low-risk) and advanced cSCC, according to the European guidelines (reproduced with permission from [2]; published by the European Journal of Cancer, 2020). In addition, it is noted that since the publication of this figure, anti-PD-1 agent pembrolizumab has been approved by US FDA for patients with recurrent or metastatic cSCC that is not curable by surgery or radiation. EGFRi: EGFR inhibitors, RT: radiotherapy; ^a^ For detailed indications and recommendations of treatment, refer to relevant section text in the European guidelines; ^b^ Locally advanced, by definition not amenable to curative surgery or curative RT; ^c^ Lymph node dissection as indicated; ^d^ All systemic treatments are off-label, except for anti-PD-1 agent cemiplimab that is approved by FDA/EMA for patients with locally advanced or metastatic cSCC who are not candidates for curative surgery or curative radiation.

Cutaneous squamous cell carcinoma is the second most common skin cancer, and accounts for 20% of keratinocyte carcinomas [1]. It may be in situ or invasive. In this review, we will address invasive cutaneous squamous cell carcinoma (referred hereafter as cSCC). Invasive cSCC is classified as either primary common cSCC or as advanced cSCC. Primary common cSCC represents the majority of cSCCs and refers to localized cSCC with no metastasis at presentation, overall having very good prognosis and 5-year cure rates exceeding 90%. In turn, primary common cSCC is further classified as low-risk cSCC (more commonly) or as high-risk cSCC, based on the risk for recurrence and metastasis [1]. Advanced cSCC is classified as locally advanced (lacSCC), or metastatic (mcSCC) with locoregional and/or distant metastasis. LacSCC is defined as non-metastatic cSCC, not amenable to either surgery or radiotherapy with reasonable hope for cure, because of multiple recurrences, large extension, bone erosion or invasion, or deep infiltration beyond subcutaneous tissue into muscle or along nerves, or else tumors in which curative resection would result in unacceptable complications, morbidity, or deformity (Figure 1) [1].

The focus of this review is on the recent advances in the diagnosis and management of high-risk cSCC. The interest in high-risk cSCC relies on its higher risk of progression to advanced cSCC, as it represents the main pool of cSCCs that give rise to advanced tumors [2]. In cSCCs overall, the proportion of local recurrence is approximately 3–5% and the proportion of nodal metastasis is approximately 3–5% [2]. It is noteworthy that these risks may be considerably higher in high-risk cSCC, with a frequency of local recurrence and metastasis reaching 30% [2,3,4,5,6,7]. However, it has been shown that, in addition to features of the tumor, the length of follow-up time and the treatment used may impact the risk of local recurrence and metastasis [8].

## 2. Definition of High-Risk cSCC

High-risk cSCC is defined as invasive cSCC, staged as N0 (without detectable regional lymph nodes) and M0 (without distant metastasis), that has features associated with a higher risk for local recurrence and metastasis [1]. An appropriate staging will confirm the diagnosis of localized cSCC and document the absence of locoregional or distant metastasis. Staging systems for cSCC include the AJCC eighth edition staging (American Joint Committee on Cancer, 2017) [9], the UICC eighth edition (Union for International Cancer Control, 2017) [10], and the Brigham and Women’s Hospital T classification system (BWH) [11]. A presentation of the staging systems for cSCC is beyond the scope of this article and may be found in the European guidelines [1].

**Figure 2:**
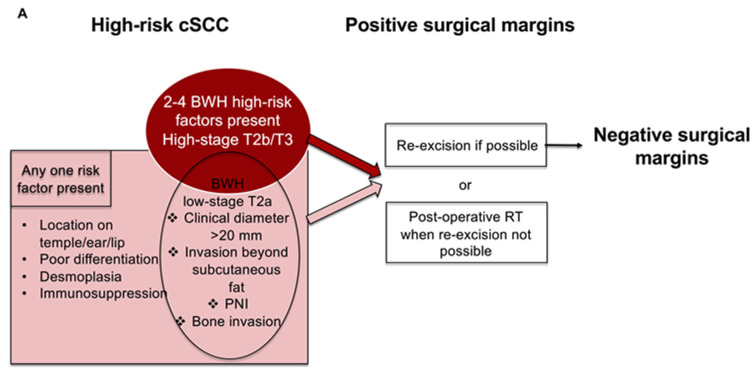

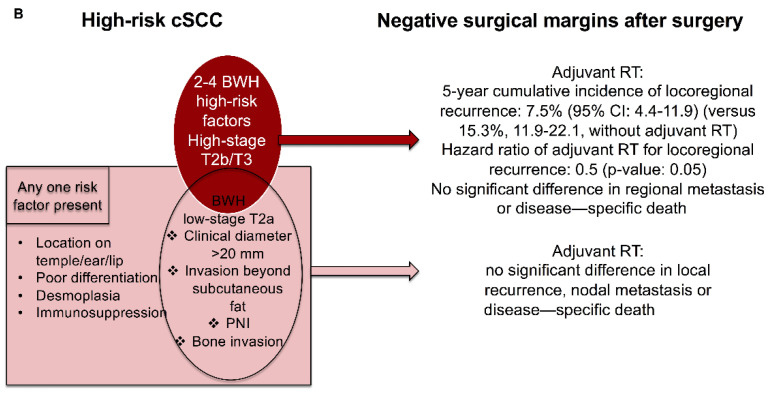
(**A**) Management of high-risk cSCC with positive surgical margins based on current guidelines. (**B**) Results from recently published studies on the use of adjuvant RT after surgery of high-risk cSCC with negative (clear) surgical margins [44,46,47,48,49].

In the European guidelines, there are eight proposed intrinsic high-risk factors for local or metastatic recurrence, including patient- or tumor-related features, and one extrinsic high-risk factor. These eight intrinsic high-risk factors include clinical diameter > 20 mm, localization on temple/ear/lip, thickness > 6 mm or invasion beyond subcutaneous fat, poor grade of differentiation, desmoplasia, microscopic symptomatic or radiological PNI, bone erosion, and immunosuppression. The presence of positive surgical margins is an extrinsic high-risk factor [1]. Table 1 details the high-risk factors that are proposed in the current European, British Association of Dermatology (BAD), and NCCN guidelines. The different sets of high-risk factors included in the current guidelines reflect the need for further evidence associating high-risk features with prognosis. Currently, there are mostly retrospective studies available, including heterogeneous groups of patients and each assessing different risk factors and prognostic outcomes (local recurrence (LR), nodal metastasis (NM), distant metastasis, disease-specific survival (DSS), or overall survival (OS)). Further complexity in accurately defining high risk stems from the fact that the presence of more than one risk factor may significantly increase risk for worse prognosis. This concept of the combination of high-risk features was introduced in the BWH classification system for the T stage [11]. The BWH system includes four high-risk factors in addition to bone invasion: (1) poor differentiation, (2) PNI (of any caliber initially [11], and ≥0.1 mm in the modified BWH staging system [7]), (3) diameter ≥ 2 cm (in contrast to AJCC8 which uses a cut-off for diameter of >2 cm), and (4) invasion beyond subcutaneous tissue. BWH-T1 tumors are defined as cSCC with none of these risk factors. BWH-T2 tumors are defined as low-stage T2a (with one risk factor) or high-stage T2b (tumors combining 2–3 risk factors) or T3 (tumors with all four risk factors, or have bone invasion) [7,11]. For BWH low-stage T2a cSCC, the risk of regional metastasis and disease-specific death was shown to be 5.2% and 1.2%, respectively, while for BWH high-stage T2b/T3, the risk was 25% and 19%, respectively [4]. Despite the overall variability in risk factors, there are similar high-risk factors in the NCCN, BAD, and European guidelines to highlight localized cSCCs with a higher risk for poorer prognosis (Figure 2).

Another relevant issue is that different high-risk factors may not influence all prognostic outcomes, such as the risk of local recurrence, nodal metastasis, or disease-specific death [14,15]. This is important as nodal metastasis and disease-specific death are major prognostic outcomes. Table 2 shows the risk factors significantly associated with local recurrence, in the meta-analysis of Thompson et al., 2016, and the risk factors associated with disease-specific death in cSCC that was localized at initial presentation, in the meta-analysis of Dessinioti et al., 2022.

Two nationwide nested case-control studies included 1104 metastatic cSCCs and showed that independent risk factors for metastasis included diameter, thickness, poor differentiation, invasion in/beyond subcutaneous fat, male sex, perineural/lymphovascular invasion, and facial localization [27].

A meta-analysis on risk factors associated with disease-specific death in localized cSCC showed that only immunosuppression conferred a higher risk. However, there was a small number of studies reporting disease-specific death, and the risk factors were variably defined across studies [15]. A nationwide cancer registry study in 11,137 patients with cSCC reported that, among the 71 cSCC-specific deaths, 39 patients did not have metastases, highlighting a group of non-metastatic cSCC leading to disease-specific death [28]. Similarly, Eigentler et al. reported that disease-specific death occurred in 70% of patients due to either local infiltration by the tumor or nodal infiltration. The remaining 30% of patients died due to visceral metastases (30%) [19]. These findings underscore the risk of death in patients with non-metastatic localized cSCC, possibly due to local complications and underlying tissue destruction. This current gap in knowledge on the causes of disease-specific death in patients with localized cSCC, which needs to elucidated in the future, was highlighted in a meta-analysis of disease-specific death in cSCC that was localized at initial diagnosis [15].

Furthermore, a 40-gene expression profile (GEP) test was developed and validated for predicting the risk for metastasis in localized, high-risk cSCC [29]. The 40-GEP was further validated and showed significant prognostic value in a multicenter study in 420 primary cSCCs. The combination of 40-GEP results with clinicopathological risk factors improved the metastatic risk classification of cSCCs [30].

## 3. Diagnosis of High-Risk cSCC

After the histological confirmation of cSCC, it is recommended that the patient undergoes a physical examination, including the skin and nodal basins. The primary site of cSCC should be carefully evaluated for the presence of in-transit cutaneous metastasis. A palpation of the regional nodal basins is important to exclude the presence of palpable lymph node metastasis. Also, a full-body skin examination is conducted for the presence of a potential additional skin cancer.

In addition, the European guidelines propose to consider imaging for high-risk cSCC without palpable lymph nodes to rule out subclinical nodal metastasis [1]. Similarly, the British guidelines propose to consider high-resolution ultrasound of the regional nodes in the clinically N0 setting for very-high-risk lesions, such as pT2 or greater lip cSCC [13].

There is limited data on imaging for high-risk cSCC and prospective studies are needed to determine best practices. In a retrospective study in 246 high-risk HNcSCCs, who underwent baseline ultrasonographic imaging of their lymph nodes, this was more sensitive (sensitivity 91%, specificity 78%) than clinical examination alone (sensitivity 50%, specificity 96%) for the detection of lymph node metastasis. Regarding patients with a negative clinical examination, 9 of 11 metastases were detected by ultrasonography, with 82% sensitivity and 79% specificity and positive predictive value of 17%. It was suggested that while there was high sensitivity of the ultrasound for surveillance detection of nodal metastases, there was the possible limitation of the high rate of false-positive findings, requiring FNAC biopsy. The criteria used for suspicious lymph nodes on ultrasonography were a short axis larger than 5 to 6 mm, round shape, absence of fatty hilium, or extranodal extension [31]. Imaging in BWH high-stage cSCC altered the management in 33% of patients [32].

Sentinel lymph node biopsy (SLNB) is a technique that has been used to identify occult metastases in lymph nodes draining the tumor site when there is no clinical or radiological suspicion of involved lymph nodes. Preoperative lymphoscintigraphy using technetium-labeled sulfur colloid is performed. The SLN is located with the use of lymphoscintigraphy before the procedure and with a hand-held gamma probe. Intraoperative blue dye injection may assist in node identification [33]. Submitted candidate sentinel lymph nodes are step-sectioned and examined with light microscopy with conventional H&E staining. Some samples undergo additional immunohistochemical staining using a pancytokeratin marker [34].

In the European guidelines 2020, SLNB was not recommended for cSCC outside the setting of clinical trials [1]. The potential benefits from SLNB on patient selection and earlier treatment of regional metastases in adjuvant clinical trials have led to increased interest in SLNB, but its value in the management and outcome of patients with high-risk tumors is currently unknown. The variability of definitions given for high-risk cSCC warranting SLNB, the rarity of nodal metastasis at first diagnosis of cSCC, and lack of long-term follow-up in available studies on SLNB has hindered the identification of patients, most likely to benefit from this procedure [35]. No studies were found showing the prognostic utility of positive SLNB after adjustment for confounding factors, and the criteria for recommending it varied considerably from study to study. Overall, their results report worse survival for those with a positive SLNB, as expected for tumors that have metastatic spread. Another limitation is the higher false-negative rate in SLNB due to the anatomical diversity of the lymphatic drainage of the head and neck, where the majority of cSCCs are located. The overall frequency of a positive SLNB in patients with cSCC was 8% in the systematic review of Tejera-Vaquerizo et al., in 2018 [36], and 12.3% in the meta-analysis of Schmitt et al. [36]. The positivity of SLNB depended on the tumor stage, and while it was 0% in AJCC T1 tumors, it was 60% in AJCC T4 tumors. Similarly, by the BWH classification, the positivity of SLNB was 7.1% (6/85) in low-stage BWH T2a, and increased to 29.4% and 50% in high-stage T2b and T3 tumors, respectively [36].

## 4. Primary Treatment of High-Risk cSCC

Surgery is the primary treatment for cSCC. The primary goals of treatment are clearance of the tumor, and the preservation of function and cosmesis [37]. When patients are not eligible to undergo surgery, e.g., locally advanced disease or frail elderly patients with comorbidities, radiotherapy (RT) may be considered as a primary treatment [37]. Primary treatment recommendations for high-risk cSCC in current guidelines are summarized in Table 3.

It is important to note that surgical excision is considered the first-line treatment for resectable primary cSCC and aims at clinical and microscopic complete resection (R0 surgery) with clear (negative) histological margins [38]. The European guidelines suggest a 5 mm clinical safety margin for low-risk lesions and a 6–10 mm clinical safety margin or micrographically controlled surgery for high-risk cSCC (Figure 1) [37]. An excision with histological control of peripheral and deep-excision surgical margins is recommended as standard therapy [37]. Micrographically controlled surgery (MCS) refers to the technique of surgical excision that includes processing skin tissue in horizontal sections and examining them under a microscope. The process is repeated until no cancer is identified at the surgical margins, if anatomically possible. MCS include Mohs micrographic surgery (MMS) and 3D histology [39]. In MMS, frozen sections are used, while 3D histology uses paraffin sections with diverse modifications of sectioning the tissue specimen [40]. The peripheral and deep en face margin assessment (PDEMA) technique is the method recommended in the NCCN guidelines. It is defined as a descriptive term for surgical techniques that perform high-quality histologic visualization and complete margin assessment, including the complete deep and peripheral margin [12]. PDEMA techniques, apart from Mohs, include the Tubingen muffin technique and the Tubingen torte technique [12,41]. The meta-analysis of Fraga et al. [32] compared recurrence for complete margin assessment versus excision with sectional assessment in high-risk keratinocyte carcinomas. They reported significantly lower locoregional recurrences with complete margin assessment versus sectional assessment for all keratinocyte carcinomas and for cSCC with PNI. The systematic review of Lansbury et al. found no randomized trial comparing MMS with conventional surgical excision for cSCC [42].

Across current guidelines, there are different sizes for safety margins recommended for high-risk cSCC; however, it has been widely accepted that wider safety margins are needed for high-risk cSCC compared to low-risk tumors. The recommendations on the size of surgical margins are mainly based on expert consensus and retrospective and observational studies, and there are no studies comparing directly different safety margins. It is of key importance to secure negative (clear) surgical margins whenever possible. A re-excision is recommended in the case of positive margins, when feasible (Table 3).

Regarding primary radiotherapy compared to other local treatment modalities, there are no prospective randomized trials studying the efficacy in local tumor control and patient survival. The meta-analysis of Lansbury et al. reported a pooled local recurrence rate of 6.4% with external radiotherapy for cSCC, but high-risk cSCC was not studied separately [42]. The systematic review of Krausz et al. on radiation therapy for cSCC highlighted that high-risk features were not described and prognosis was not reported separately for each radiotherapy type (primary or adjuvant/salvage) [43].

## 5. Post-Operative RT for High-Risk cSCC with Residual Disease after Surgery

After surgery, for high-risk cSCC with positive surgical margins, the recommendations are clear and re-excision is recommended whenever possible in order to achieve negative margins. When re-excision is not possible, post-operative radiotherapy is recommended [37,44] (Figure 2a). Recently, Revelles-Penas et al. showed that cSCC with microscopic residual disease after surgery had a lower risk of local recurrence after post-operative RT to the tumor bed compared with observation, but there was no difference for nodal metastasis and disease-specific death [45].

## 6. Adjuvant Treatment for High-Risk cSCC with Negative Surgical Margins

The benefit of adjuvant treatment after complete resection with negative surgical margins (R0) of high-risk cSCC remains inadequately established. As a result, the recommendations by current guidelines regarding a possible benefit with adjuvant treatment for high-risk cSCC with negative surgical margins after surgery are not uniform (Table 4).

Recently published results comparing adjuvant RT to surgery alone have accumulated in patients with high-risk cSCC with clear surgical margins [44,46,47,48,50]. In the meta-analysis of Kim et al., in high-risk non-metastatic cSCC (any high-risk factor present) treated with margin-negative resection (29 retrospective, 2 prospective, 2 case series), there were no statistically significant differences in poor outcomes between surgery only and surgery with adjuvant RT [46]. On the other hand, the meta-analysis of Zhang et al. reported lower recurrence, longer disease-free survival, and longer overall survival with adjuvant radiotherapy, but included primary as well as metastatic cSCC, and the benefit of adjuvant RT may have concerned nodal metastatic cSCC [50]. In a retrospective study of 882 high-risk cSCCs treated with MMS, there was no significant difference in progression-free survival in patients treated with Mohs alone compared to patients treated with adjuvant therapy, but matching was imperfect [47].

Similarly, the matched retrospective study of Ruiz et al. on adjuvant RT for primary cSCC with clear surgical margins reported no significant difference in local recurrence, metastasis, or disease-specific death with adjuvant RT versus surgery alone. Interestingly, there was no significant difference with adjuvant RT in these outcomes in a subgroup of cSCC with large-caliber nerve invasion that had clear surgical margins. The authors proposed that adjuvant RT did not improve outcomes compared to surgery alone due to a low baseline risk for poor outcomes in primary cSCC with clear histologic margins [48].

There are studies showing a benefit with adjuvant RT compared to surgery alone for cSCC with multiple high-risk factors. Ruiz et al. [49], focusing in high-stage BWH-T2b/T3 cSCCs (with 2–4 high-risk factors) with clear surgical margins, showed that adjuvant RT halved the 5-year cumulative incidence of locoregional recurrence compared to surgery alone (7.5%, 95% CI: 4.4–11.9, versus 15.3%, 95% CI: 11.9–22.1, respectively). However, the hazard ratio of adjuvant RT for locoregional recurrence was marginally not statistically significant (*p*-value: 0.05), and there was no significant difference for regional metastasis or disease-specific death [49].

It seems that it is difficult to discern the group of high-risk cSCCs that may benefit from adjuvant RT, as a universal beneficial effect for a cSCC with any high-risk factor resected with clear surgical margins has not been established (Figure 2b).

There are no solid data to support the use of adjuvant systemic treatment in localized cSCC after RO resection [37,51,52,53,54,55,56]. There are ongoing clinical trials of anti-PD-1 immunotherapy as adjuvant treatment for high-risk cSCC after surgery and radiation therapy [57,58].

## 7. Referral of Patients with High-Risk cSCC to Multidisciplinary Tumor Board

In the NCCN guidelines 2022, it is recommended to refer patients with high-risk/very-high-risk cSCC, with positive margins after surgery when re-excision is not feasible, to multidisciplinary tumor board meeting to discuss the possible options (re-excision if feasible, or combination of systemic therapy with RT). Further, a multidisciplinary consultation is recommended to consider adjuvant RT for high-risk/very-high-risk cSCC with negative margins, if there is extensive perineural, larger, or named nerve involvement, or if other poor prognostic features are present [12].

In the British guidelines, it is recommended to refer to specialist skin cancer multidisciplinary tumor board meetings (SSMDT) in order to consider Mohs in selected people with cSCC. Additionally, a multidisciplinary tumor board meeting is recommended for cSCC with one or more involved margins to review the histology, to discuss cSCC with symptomatic PNI and/or radiological PNI, to discuss cSCC considered for RT with a clinical/radiation oncologist present, and to discuss further management of high-risk cSCC previously removed by curettage and cautery [13].

## 8. Follow-Up of Patients with High-Risk cSCC

There is currently no standardized follow-up schedule for patients with cSCC. The rationale for follow-up is to detect a possible recurrence of cSCC, as well as to monitor the patient for the possible development of a new primary skin cancer. Patients with high-risk cSCC should be informed on the frequency of follow-up in the clinic and encouraged to perform skin self-examination. Special consideration is advised for immunosuppressed patients, including the need for lifelong follow-up, and referral to dedicated clinics with relevant experience, when possible [1].

Factors to consider regarding the frequency and intensity of the follow-up schedule include the risk and possible time of metastasis development. Most metastatic cSCCs are diagnosed within 2 years of the primary cSCC [28,59]. A nationwide cancer registry study showed that, from all mcSCCs, 74% resulted from the first cSCC and 26% arose from subsequent cSCCs [28]. High-risk cSCC has increased risk of nodal metastases, which was quantified based on the high-risk features in the meta-analysis of Thompson et al., reporting a 11-fold risk for invasion beyond subcutaneous fat, 10-fold risk for thickness > 2 mm, 7-fold risk for thickness >6 mm, 6-fold risk for diameter > 20 mm, 5-fold risk for poor differentiation, 3-fold risk for PNI, and a 3-fold risk for location on temple [14]. The risk is further modified by the number of high-risk factors present, as reported in the Brigham and Women’s Hospital (BWH) classification system. In BWH classification, high-stage cSCC (T2b/T3) accounted for 70% of nodal metastasis (with 2–4 risk factors present) [7]. Another study offered patients with BWH high-stage cSCC imaging at baseline and then every 4–6 months for 2 years. The authors reported that a majority (56%) of detections were not seen initially but rather during surveillance imaging in the 2 years post-treatment. In the follow-up imaging cohort, imaging identified nodal metastasis in 19% of patients that was not palpable on clinical examination [60].

In the European guidelines, it is proposed that patients with high-risk cSCC are followed up every 3–6 months for the first 2 years, and every 6 months for years 3 to 5, and annually thereafter. Also, depending on individual risk findings and the number of risk factors present, a regional lymph node ultrasound may be advised every 3 to 6 months in the first 2 years [1,3].

## 9. Conclusions

The assessment of risk is particularly relevant for common cSCC to identify the few with a high risk of local recurrence, metastasis, or disease-specific death among all other low-risk tumors. These findings highlight high-risk cSCCs as a main group of tumors for which timely diagnosis and effective treatment may halt their further progression and aim to prevent and lower the incidence of advanced cSCCs. This is particularly important given the lower survival rates of advanced cSCCs, the need of systemic treatments for locally advanced or metastatic cSCC, and considering that a group of patients will not respond to systemic treatments [2,38].

The ascertainment of prognostic factors defining high-risk cSCC may have an impact on further management, with appropriate surgical treatment, ensuring negative surgical margins whenever possible, the consideration for possible post-operative or adjuvant radiotherapy, depending on the status of surgical margins and high-risk factors present, and more regular follow up. The accumulation of results from well-designed studies over the years has led to progress in the definition, the diagnosis, and the management of high-risk cSCC. Future studies on current gaps in knowledge may provide further evidence to guide recommendations and best clinical practices for patients with high-risk cSCC.

## Figures and Tables

**Table 1:** Risk factors for high-risk cSCC in current guidelines are summarized. The presence of any high-risk factor places the patient in the high-risk category.

High-Risk Factors	European 2020 [1]	US NCCN 2022 [12]		UK BAD 2020 [13]	
	High Risk for Recurrence (Local or Metastatic)	High-Risk Factor for Local Recurrence, Metastasis, or Disease-Specific Death	Very High Risk for Local Recurrence, Metastasis, or Disease-Specific Death	High Risk for Local Recurrence, Nodal Metastasis, Or Disease-Specific Death	Very High Risk for Local Recurrence, Nodal Metastasis, Or Disease-Specific Death
**Tumor-Related High-Risk Factors**					
Tumor diameter	>20 mm	Trunk, extremities >2 cm–≤4 cm	>4 cm any location	>20–40 mm	>40 mm
Localization	On temple/ear/lip	Head, neck, hands, feet, pretibial, and anogenital (any size)		On ear/lip	-
Thickness	Thickness > 6 mm or Invasion beyond subcutaneous fat	-	>6 mm or Invasion beyond subcutaneous fat	Thickness > 4–6 mm	Thickness > 6 mm
Invasion				Invasion into subcutaneous fat	Invasion beyond subcutaneous fat
Differentiation	Poor grade differentiation	-	Poor grade differentiation	Poor grade differentiation	-
Histological feature	Desmoplasia	Acandtholytic (adenoid), adenosquamous, or metaplastic (carcinosarcomatous)	Desmoplasia	Lymphovascular invasion	High-grade histological subtype—adenosquamous, desmoplastic, spindle/sarcomatoid/metaplastic
Perineural invasion (PNI)	Histological/symptomatic/radiological PNI	Yes	PNI of a nerve lying deeper than the dermis or measuring ≥ 0.1 mm	Perineural invasion—dermal only; nerve diameter < 0.1 mm	Perineural invasion present in named nerve; nerve ≥0.1 mm; or nerve beyond dermis
Lymphatic or vascular involvement	-	-	Yes	-	-
Bone erosion/invasion	Bone erosion	-	-	-	Any bone invasion
Tumor on scar/chronic inflammation/RT	-	Site of prior RT or chronic inflammation	-	Tumor arising within scar or area of chronic inflammation	-
In-transit metastasis	-	-	-	-	In-transit metastasis
Borders	-	Poorly defined	-	-	-
Primary vs recurrent	-	Recurrent	-	-	-
Rapidly growing tumor	-	Yes	-	-	-
Neurologic symptoms	-	Yes	-	-	-
**Patient-Related Risk Factors**					
Immunosuppression	Yes	Yes	-	Iatrogenic IS or biological therapies, frailty and co-morbidities, HIV, HAART	As for high risk, especially SOTRs, hematological malignancies, such as CLL or myelofibrosis, other significant IS
**Extrinsic Risk Factors**					
Positive margins	Yes	-	-	One or more involved or close margin in a pT1 tumor. Close margins in a pT2 tumor.	One or more involved or close margin in a high-risk tumor.
Grade of recommendation	B (recommendation)	-	Category 2A (lower-level evidence, uniform NCCN consensus)	GPP (informal consensus)	GPP (informal consensus)

**Table 2:** Risk factors for local recurrence, nodal metastasis, and disease-specific death in meta-analyses. Statistically significant risk ratios are marked in bold.

Risk Factor	Thompson, 2016 [14]				Dessinioti, 2022 [15]	
	Local Recurrence	Nodal Metastasis	Disease-Specific Death		Disease-Specific Death in Localized cSCC at Presentation	
	Risk Ratio (95% CI)	Risk Ratio (95% CI)	Included Studies	Risk Ratio (95% CI)	Included Studies	Risk Ratio (95% CI)
Poor differentiation	**2.66 (1.72–4.14)**	**4.98 (3.30–7.49)**	Brinkman et al. [16] Friedman et al. [17] Karia et al. [7] Kyrgidis et al. [18]	**5.65 (1.76–18.20)**	Brinkman et al. [16] Eigentler et al. [19] Karia et al. [7] Ruiz et al. [20]	3.72 (0.80–17.28)
Depth beyond fat	**7.61 (4.17–13.88)**	**11.21 (3.59–34.97)**	Clayman et al. [21] Friedman et al. [17] Karia et al. [7] Kyrgidis et al. [18]	**4.49 (2.05–9.82)**	Conde-Ferreiros et al. [22] Karia et al. [7] Ruiz et al. [20]	2.24 (0.34–14.75)
Diameter 20 mm or more	**3.22 (1.91–5.45)**	**6.15 (3.56–10.65)**	Karia et al. [7]	**19.10 (5.80–62.95)**	Karia et al. [7] Ruiz et al. [20]	4.57 (0.20–106.66)
PNI present	**4.30 (2.80–6.60)**	**2.95 (2.31–3.75)**	Clayman et al. [21] Kyrgidis et al. [18] Schmults et al. [23]	**4.06 (3.10–5.32)**	Schmults et al. [23] Ruiz et al. [20]	1.63 (0.21–12.88)
Thickness ≥ 6 mm	**7.13 (3.04–16.72)**	**6.93 (4.02–11.94)**	-	-	Conde-Ferreiros et al. [22] Eigentler et al. [19]	2.44 (0.30–19.66)
Thickness (continuous)	-	-	-	-	Tschetter et al. [24]	1.20 (1.00–1.44)
Thickness > 2 mm	**9.64 (1.30–71.52)**	**10.76 (2.55–45.31)**	-	-	-	-
Location ear	1.28 (0.56–2.90)	**2.33 (1.67–3.23)**	Griffiths et al. [25] Schmults et al. [23]	**4.67 (1.28–17.12)**	Eigentler et al. [19] Griffiths et al. [25] Schmults et al. [23]	1.71 (0.61–4.78)
Location lip	1.28 (0.56–2.90)	**2.28 (1.54–3.37)**	-	**4.55 (1.41–14.69)**	-	-
Location head/neck	-	-	-	-	Schmults et al. [23] Ruiz et al. [20]	0.98 (0.29–3.24)
Location temple	**3.20 (1.12–9.15)**	**2.82 (1.72–4.63)**	-	1.80 (0.22–14.79)	-	-
Immunosuppression	1.51 (0.81–2.81)	**1.59 (1.07–2.37)**	Karia et al. [7]	0.35 (0.05–2.58)	Eigentler et al. [19] Karia et al [7] Ruiz et al. [20] Tam et al. [26]	**1.85 (1.32–2.61)**

-: not reported. Statistically significant risk ratios are shown in bold.

**Table 3:** Recommendations for the management of high-risk cSCC in current guidelines.

Treatment for High-Risk cSCC	European 2020 [37]	US NCCN 2022 [12]	UK BAD 2020 [13]
Surgery	As first-line treatment: excision with histological control aiming at R0 excision (GOR: A)	Mohs or other forms of PDEMA (preferred for very high risk) Or standard excision with wider surgical margins and postoperative margin assessment (GOR: 2A)	Offer standard surgical excision as first-line treatment for resectable primary cSCC (GOR: Strong)
	Standard excision with histological confirmation of peripheral and deep margins or MMS/MCS (GOR: B)		Consider MMS in selected cSCC after SSMDT (GOR: Weak)
Clinical safety margins	6–10 mm (GOR: B)	Wider than 6 mm (GOR: 2A)	≥6 mm for high risk ≥10 mm for very high risk (GOR: Strong)
Primary RT	Primary RT should be considered as an alternative to surgery for inoperable or difficult-to-operate tumors or in the absence of consent to surgical excision (GOR: B)	Primary RT +/− systemic therapy, as an alternative to surgery for non-surgical candidates (GOR: 2A)	Offer to selected people with cSCC as an option after MDT Offer when surgery is not feasible or would be challenging or likely to result in an unacceptable functional or aesthetic outcome (GOR: Strong)
	-	-	Consider primary RT for locally recurrent cSCC (GOR: GPP)
	-	-	Consider conformal RT including the entire course of the involved nerve in people with cSCC with symptomatic PNI and/or radiologic evidence of PNI when surgery is inappropriate (GOR: Weak)
Systemic Therapy	-	RT +/− systemic therapy for high-risk/very-high-risk cSCC, for non-surgical candidates. Discuss in multidisciplinary consultation, RT +/− systemic therapy for high-risk/very-high-risk cSCC with positive margins if re-excision not feasible.	-

PDEMA: Peripheral and deep en face margin assessment, GOR: grade of recommendation, GPP: good practice point, MMS: Mohs micrographic surgery, MCS: micrographically controlled surgery, RT: radiotherapy, SSMDT: specialist skin cancer multidisciplinary tumor board meetings, -: a recommendation is not given.

**Table 4:** Recommendations on adjuvant therapy for high-risk cSCC in current guidelines.

Adjuvant Therapy	European 2020 [37]	US NCCN 2022 [12]	UK BAD 2020 [13]
Adjuvant radiotherapy	Post-operative RT should be considered after surgical excision for cSCC with positive margins and re-excision not possible	Recommend multidisciplinary consultation and consider adjuvant RT, for local, high-risk/very-high-risk cSCC with negative margins, if extensive perineural, larger, or named nerve involvement, or if other poor prognostic features. Noted that the outcome of adjuvant RT following resection of any cSCC with negative surgical margins is uncertain (GOR: 2A)	Offer adjuvant RT to people with incompletely excised cSCC, where further surgery is not possible and in those at high risk for local recurrence (PNI [multifocal, named nerve, and/or diameter of nerve >0.1 mm, below the dermis], immunosuppression or recurrent disease) (GOR: Strong)
-			Consider adjuvant RT for completely excised T3 tumors, with multiple high-risk factors including >6 mm thickness and invasion beyond subcutaneous fat (GOR: Weak)
			Consider adjuvant RT for locally recurrent cSCC (GOR: GPP)
			Do not offer post-operative RT for people with completely excised T1 or T2 cSCC and with microscopic, dermal only, nerve diameter < 0.1 mm PNI (GOR: Strong against)

GOR: grade of recommendation.

## Data Availability

The data presented in this study are available in the article.
